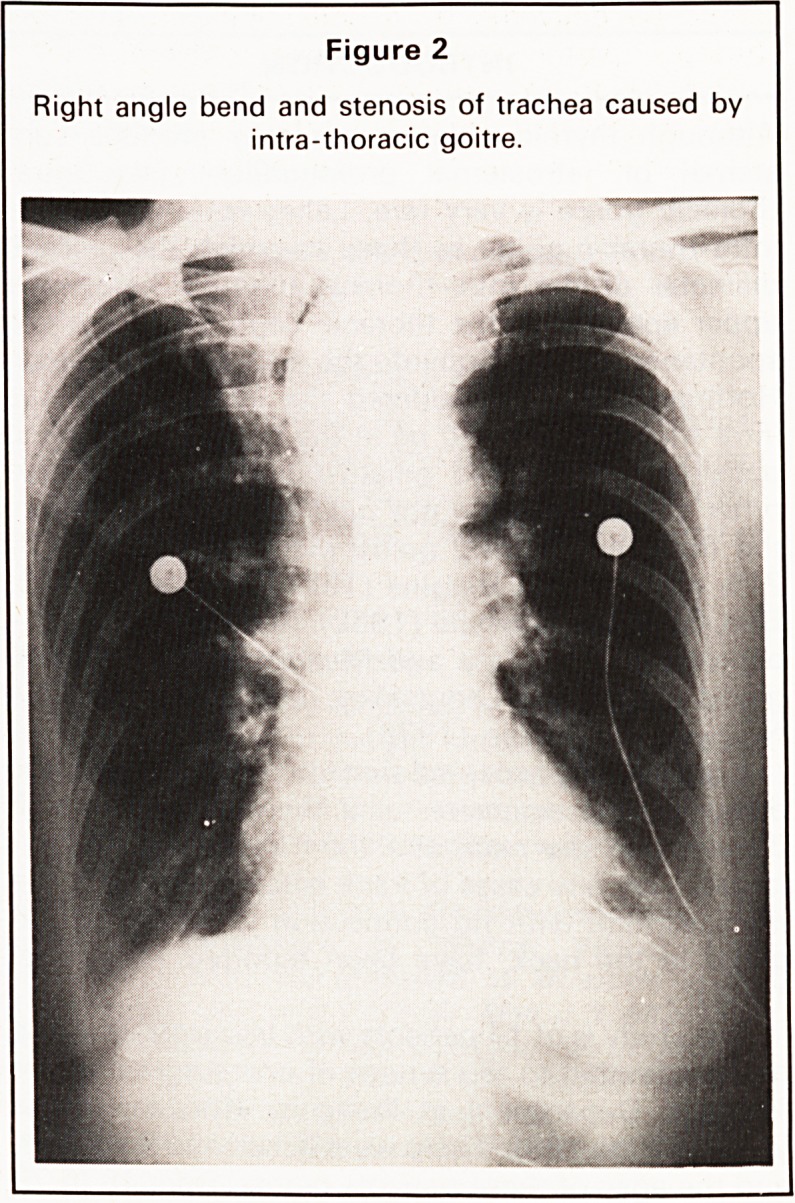# Intra-Thoracic Goitre with Life Threatening Complications: A Continuing Diagnostic Problem

**Published:** 1984-01

**Authors:** E. Andrianopoulos, G. Keen

**Affiliations:** Frenchay Hospital, Bristol; Frenchay Hospital, Bristol

## Abstract

Fourteen patients underwent surgery for benign intra-thoracic goitre many presented with respiratory distress due to tracheal compression, and in some desperately ill patients inoperable cancer of the lung was diagnosed. The plain chest X-ray revealed the goitre and once the true diagnosis was suggested, prompt surgical intervention followed.


### Intra-thoracic Goitre with Life Threatening Complications: a Continuing Diagnostic Problem

**E. Andrianopoulos, M.D., M.S.**
**G. Keen, M.S., F.R.C.S.**
Frenchay Hospital, Bristol

### SUMMARY

Fourteen patients underwent surgery for benign intra-thoracic goitre many presented with respiratory distress due to tracheal compression, and in some desperately ill patients inoperable cancer of the lung was diagnosed. The plain chest X-ray revealed the goitre and once the true diagnosis was suggested, prompt surgical intervention followed.

### INTRODUCTION

Although thyroid enlargement may produce substernal or retrosternal prolongation, true intra-thoracic goitre is very rare. Lahey in 1945 defined intra-thoracic goitre as those in which the greatest diameter of the intra-thoracic mass is below the upper aperture of the thoracic cage, and in which spontaneous reduction into the neck does not occur. Hoffman (1955) considered the incidence of true intra-thoracic goitre to be in the order of 0.2–3% of patients with thyroid enlargement. If we include other cases which do not fulfil the above criterion, the frequency among goitre patients is as high as 21% according to Higgins (1927), McCort (1949), Ellis, Good and Seybold (1952), Sherman and Shahbahrami (1966), Tala and Maamies (1967), Georgiadis, Katsas and Leoutsakos (1970) and Lesavoy, Norberg and Kaplan (1975).

It is generally accepted that intra-thoracic goitre is a mediastinal extension of a thyroid gland which develops in the neck and then descends and enlarges. Very few cases of frank ectopic thyroid in the mediastinum, with no connection with the thyroid gland in the neck, have been reported (Doundas, 1964).

This study is of 14 patients with true intra-thoracic goitre admitted to the service of one surgeon in the Thoracic Department at Frenchay Hospital in the years 1970 to 1982. There were 8 men and 6 women, and the ages of these patients ranged from 48 to 79 years, with an average of 60.7 years. All patients had been previously seen by physicians or other surgeons, and some presented important diagnostic difficulties. The tumour in all 14 cases was lying in the upper or anterior mediastinum. In 8 patients (6 men and 2 women), the tumour originated from the right lobe of the thyroid and lay in the right anterior mediastinum, in 5 patients (3 women and 2 men) it was in the left anterior mediastinum originating from the left lobe of the thyroid gland, and in one female patient the tumour originated from the left lobe of the thyroid gland but extended posteriorly into the right anterior mediastinum. The weight of the removed tumours varied from 100 to 420 g. and the size from 3×6×4 cm to 14×10×6 cm. In 11 of these patients the greatest diameter of the tumour was below the thoracic inlet and in 3 it was at the thoracic inlet.

### SYMPTOMS AND SIGNS

The symptoms and physical signs of the patients are noted in Table 1. Of the 9 patients with respiratory distress, 2 were gasping for breath and near to death, and their lives were undoubtedly saved by emergency surgery. Two patients presented with severe dysphagia caused by extrinsic oesophageal pressure, and 3 further patients had non-productive cough. In 11 patients, an enlarged thyroid gland was either seen or palpated but in 3 an enlarged thyroid gland was not palpated in the neck. In 5 patients there was marked inspiratory stridor and a further 5 presented with hoarseness of the voice. Six patients had superior vena caval obstruction which was shown by dilated and enlarged veins of the neck and upper chest. In one of these patients (Figure 1) superior vena caval obstruction was so marked that when associated with a mass in both sides of the neck, and a mediastinal mass, was thought to have inoperable cancer of the lung and was treated initially with radiotherapy. One patient with superior vena caval obstruction had, in addition, Horner's

**Table 1**

**Symptoms and physical signs in 14 patients with intra-thoracic goitre**

| *Symptoms or signs* | *No. of patients* |
|---|---|
| Thyroid enlargement | 11 |
| Respiratory distress | 9 |
| SVC obstruction | 6 |
| Stridor | 5 |
| Hoarseness | 5 |
| Cough | 3 |
| Thyrotoxicosis | 3 |
| Dysphagia | 2 |
| Horner's syndrome | 1 |
| Minor stroke | 1 |

syndrome on the left side. One patient presented to the physicians with a right hemiparesis which recovered after several days and in whom a routine chest X-ray showed there to be a mass the size of an orange in the apex of the right chest. This was considered to be carcinoma of the lung with a cerebral metastasis. This patient's investigations included a thyroid scan which showed no evidence of uptake in the tumour in the right chest. He was subjected to right thoracotomy and only then was it discovered that the mass was a thyroid cyst isolated from the lung and coming into the chest on a narrow pedicle. Removal of this cyst was undertaken with difficulty from the right chest as it proved a complicated procedure to ligate safely and divide the vessels which descended from the neck. This patient suffered postoperative right recurrent nerve palsy which fortunately recovered after 1 year. His stroke also recovered completely and the patient was seen 5 years later with no residual disability, and it was considered that his stroke was an incidental association with his benign thyroid cyst.

**Figure 1**

SVC venogram showing gross dilatation and displacement of its main tributaries by the intra-thoracic goitre.

### INVESTIGATIONS

The most helpful investigation in all patients was the plain chest X-ray which revealed the presence and the extension of the mediastinal mass in all cases. The typical picture was that of a superior mediastinal pyramidal density with the base uppermost and the apex below. Tomography was undertaken in 4 patients and clarified the borders of the mass and showed its relations with adjacent important anatomical structures. The 9 patients with respiratory

**Figure 2**

Right angle bend and stenosis of trachea caused by intra-thoracic goitre.

distress had moderate to severe tracheal deviation and the 2 patients with severe stridor who required emergency surgery had severe tracheal narrowing with almost a right angle bend in both cases in the mid portion of the trachea (Figure 2). In those patients with respiratory symptoms, pulmonary function tests were very much depressed with evidence of severe airways obstruction. Thyroid scan was a useful examination and confirmed the thyroid origin of the tumour but it must be pointed out that in 2 patients, the thyroid scan failed to delineate a right sided tumour and in both of these cases right thoracotomy was undertaken. Aspiration biopsy of the tumour was performed via the neck in 2 patients, who were considered to have initially inoperable cancer of the lung. In these patients cytological examination showed degenerated thyroid tissue which pointed to the diagnosis.

### TREATMENT

All 14 patients underwent surgery. Three had been treated for thyrotoxicosis previously but were euthyroid at the time of operation. The surgical approach is seen in Table 2. In 6 of the 14 patients the tumour could be delivered by a collar incision, and in a further 4, partial sternal split down to the manubrium was required. In a further 2 patients the mediastinal tumour was so large that total sternal split was required in addition to a collar incision. In 1 patient thoracotomy alone secured the tumour which had been mis-diagnosed, and in this patient mobilisation and ligation of the pedicle resulted in a right recurrent laryngeal palsy. In a further patient in whom there was mis-diagnosis, right thoracotomy was undertaken which revealed the nature of the tumour as being a large right lobe of thyroid. In this patient the right thoracotomy was closed, the patient turned, and the thyroid secured via a collar incision.

The 2 patients with near terminal acute respiratory distress had originally been taken to the Intensive Therapy Unit preoperatively with a diagnosis of inoperable cancer. One had been treated with steroids and bronchodilators before it was considered that simple thyroid enlargement was likely. The other patient had cardiac arrest due to anoxia, and following resuscitation and tracheal intubation, recovered. Both of these patients underwent emergency surgery within 6 hours of clinical diagnosis, and in both of these patients histology showed nodular colloid goitre.

**Table 2**

**Surgical approach in 14 patients with intra-thoracic goitre**

| *Surgical approach* | *No. of patients* |
|---|---|
| Collar alone | 6 |
| Collar and partial sternal split | 4 |
| Collar and total sternal split | 2 |
| Right thoracotomy alone | 1 |
| Right thoracotomy and collar | 1 |

One patient who was being operated on through a collar incision in another hospital had the cervical portion of the mass removed but severe bleeding prevented the mediastinal extension being delivered. The wound was packed and the patient was transferred to our service, and the remainder of the tumour was removed through the same collar incision. This patient, who had suffered severe haemorrhage recovered from operation with a temporary right hemiparesis and it was assumed that cervical venous air embolism had occurred. Fortunately, this complication recovered completely within 6 months.

The extent of surgery varied. Nine patients underwent thyroid lobectomy (6 right and 3 left), and 5 had sub-total thyroidectomy. There were no deaths in this series. One female patient developed tetany which required treatment with calcium and vitamin D, although she recovered fully and these drugs were discontinued after 1 year. One patient suffered damage to the right recurrent laryngeal nerve and this was considered to be associated with the poor approach through the right chest. One female patient, who had a large intra-thoracic goitre associated with true bronchial asthma, had her recovery complicated by temporary right recurrent nerve palsy and this complication required postoperative tracheostomy. The tracheostomy tube was removed 3 months later and at the time it was noted that the vocal cords were moving normally.

### HISTOLOGY

Histological examination revealed nodular colloid goitre in 11 patients, hyperplasia of the thyroid in 2 and large cyst formation in one other. Malignancy was not seen in these patients.

### DISCUSSION

Once a goitre has descended from the neck into the mediastinum and enlarges, it will become progressively more difficult for spontaneous reduction into the neck to occur. Impaction of the tumour at the inlet of the thorax will produce progressive compression of the important structures which pass through the thoracic inlet, and those affected in decreasing order of importance are the trachea, the superior vena cava and finally the oesophagus.

Although superior vena caval compression can produce the most dramatic physical signs, it is of course tracheal compression which produces the most urgent problem and it is this symptom which if untreated will be fatal.

Several authors (Ellis et al., 1952; Judd, Beahrs and Bowes, 1960) report a higher incidence of intra-thoracic goitre in female than in male patients which is not surprising for goitre tends to be more common in women, but others (Tala and Maamies, 1967; Samaan and Murali, 1972) report that there is an equal incidence of intra-thoracic goitre in both sexes.

Although an enlarging intra-thoracic goitre tends to produce increasing symptoms, some patients may present entirely without symptoms, and the tumour is picked up accidentally at radiology or investigations for another symptom. In our series we encountered only one such patient who presented with an unassociated stroke. Most patients, however, had obvious thyroid enlargement and in these patients the lower border of the thyroid gland could not be palpated, suggesting mediastinal extension which was confirmed radiologically.

The symptoms, apart from those due to thyrotoxicosis, are those due to superior mediastinal pressure on the trachea, the oesophagus, the great vessels or the nerves. There is much speculation whether or not enlargement of the thyroid can primarily damage a recurrent laryngeal nerve although preoperative paralysis of one or either vocal cords in patients with benign thyroid enlargement is well known. It is, of course, recommended that preoperative laryngoscopy with recording of vocal cord movements is undertaken in all patients.

For anatomical reasons the more the goitre descends into the thoracic cavity, the less likely is airways' obstruction, for the trachea can then readily be displaced into the other side of the chest. Shortness of breath and stridor are the commonest complaints. Dysphagia, pain and Horner's syndrome are noted very infrequently but hoarseness is not rare. When the recurrent laryngeal nerve is paralysed, it may indicate malignant involvement of the gland.

The most useful investigation in these patients is plain chest X-ray, noting the typical features of displacement and compression of the trachea (Figure 2), and screening may show that the mass rising and falling on swallowing. Other investigations such as barium swallow, tomography, laryngoscopy and bronchoscopy are of secondary importance, and if bronchoscopy is to be undertaken in patients with stridor, it is imperative that facilities are available for immediate thyroidectomy, for this investigation may precipitate complete tracheal obstruction (Colcock, 1953; Lamke et al., 1979). Investigations such as mediastinoscopy, aspiration biopsy, venography, cardiac catheterisation and oesophagoscopy have all been undertaken but are by and large unnecessary unless it is difficult to exclude the possibility of bronchial carcinoma, aortic aneurysm, lymphoma or thymic tumour.

Once the diagnosis is confirmed, early operation is advocated and in those patients with severe symptoms operation should be undertaken as an emergency. It is widely agreed that there is no place for other than endotracheal inhalation anaesthesia, and arguments concerning the relative need for endotracheal anaesthesia versus inhalation anaesthesia with the mask merely reflected the development of pre-war surgery and anaesthesia.

The operative approach for intra-thoracic goitre varies, and we have shown that the majority of these can be removed through a collar incision with perhaps an upper sternal split. Some surgeons recommend that if the thyroid lobes are removed piecemeal or spooned out, then it is not necessary to split the sternum, but the excellent and safe exposure obtained by sternal splitting should make such ugly operations unnecessary. In those 2 patients in whom a right thyroid enlargement (undiagnosed) was approached via a right thoracotomy, the approach was very unsuitable, in 1 patient the recurrent laryngeal nerve was damaged and in the other collar incision was resorted to.

### CONCLUSIONS

Any patient with goitre and who develops stridor should be suspected of having an intra-thoracic extension which is causing compression of the trachea. Dilatation of the superficial thoracic or distension of the neck veins should give suspicion of the presence of intra-thoracic goitre and should not be necessarily regarded of sinister or malignant causation. In patients with intra-thoracic goitre presenting with acute respiratory distress, true diagnosis may be delayed as the lesion may be mistaken for an untreatable malignancy and therefore neglected. Once the true nature of the condition is clear, immediate surgical intervention is necessary, the operation and procedure of choice being a collar incision which should be extended by partial or complete sternal splitting should this be required.

### REFERENCES

COLCOCK, B. (1953) Intrathoracic goitre. *Surg. Clin. North Am.* **33**, 773–779.

DOUNDAS, P. (1964) Intrathoracic aberrant goitre. *Acta Chir.Scand.* **128**, 729.

ELLIS, F. H. Jr., GOOD, C. A. and SEYBOLD, W. D. (1952) Intrathoracic goitre. *Ann.Surg.* **135**, 79.

GEORGIADIS, N., KATSAS, A., and LEOUTSAKOS, B. (1970) Substernal goitre. *Int.Surg.* **54**, 116.

HIGGINS, C. C. (1927) Intrathoracic goitre. *Arch.Surg.* **15**, 895.

HOFFMAN, E. (1955) Intrathoracic goitre. *Br.J.Surg.* **43**, 310–314.

JUDD, E. S., BEAHRS, O. H. and BOWES, D. E. (1960) A consideration for the proper surgical approach for substernal goitre. *Surg.Gynecol.Obstet.* **110**, 90–98.

LAHEY, F. H. (1945). Intrathoracic goitres. *Surg.Clin. North Am.* **25**, 609.

LAMKE, L. O., BERGDAHL, L. and LAMKE, B. (1979) Intrathoracic goitre. A review of 29 cases. *Acta Chir. Scand.* **145**, 83–86.

LESAVOY, M. A., NORBERG, H. P. and KAPLAN, E. L. (1975) Substernal goitre with superior vena caval obstruction. *Surgery* **77**, 325–329.

McCORT, J. J. (1949) Intrathoracic goitre. *Radiology* **53**, 227.

SAMAAN, H. A. and MURALI, R. (1972) Intrathoracic goitre. *J.R.Coll.Surg.Edinb.* **17**, 45.

SHERMAN, P. H. and SHAHBAHRAMI, F. (1966) Mediastinal goitre. *Am.Surg.* **32**, 137.

TALA, P. and MAAMIES, T. (1967) Intrathoracic goitre. *Ann.Chir.Gynaecol.Fenn.* **56**, 211.

## Figures and Tables

**Figure 1 f1:**
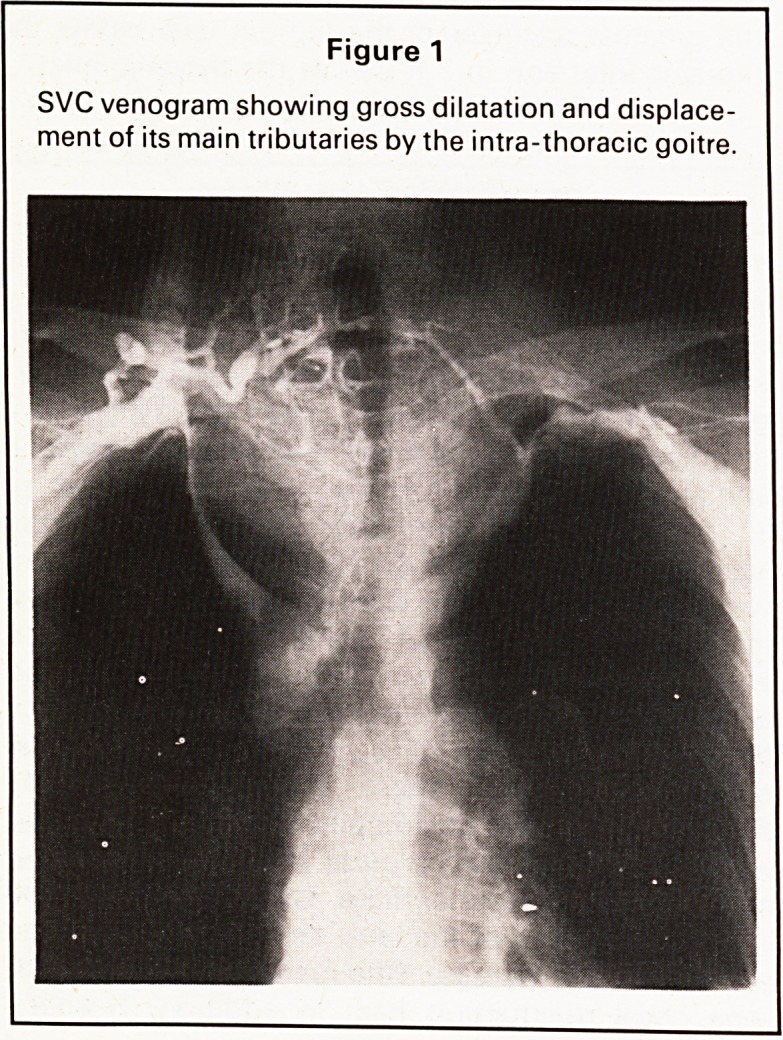


**Figure 2 f2:**